# A tutorial on bayesian multiple-group comparisons of latent growth curve models with count distributed variables

**DOI:** 10.3758/s13428-025-02624-3

**Published:** 2025-03-10

**Authors:** Jasper Bendler, Jost Reinecke

**Affiliations:** 1 Faculty of Law, University of Münster, Bispinghof 24/25, 48143 Münster, Germany; 2 Faculty of Sociology, University of Bielefeld, Universitätsstraße 25, 33615 Bielefeld, Germany

**Keywords:** Bayesian structural equation modeling, Latent growth curve models, Count data, Multiple-group growth modeling

## Abstract

Moderation effects in longitudinal structural equation models are often analysed using latent variable product terms, which can be complex and difficult to estimate, especially in large models with many panel waves. An alternative approach for categorical moderation variables is the simpler technique of multiple-group comparisons. This method allows for straightforward model specification and precise differentiation of effects in complex models. This tutorial demonstrates multiple-group comparisons using examples based on developmental trajectories of juvenile delinquency. These trajectories are modelled via a latent growth curve approach, treating the variables as count data and applying Bayesian estimation using the software M*plus*. The results are processed using the R programming language. This method addresses challenges associated with maximum likelihood estimation, particularly for latent growth models with count variables and additional exogenous latent variables. The analysis examines group differences by gender and school type in the trajectories of an unconditional growth model. It also examines the effect of legal norm acceptance on these trajectories using a conditional growth model. The moderating effects of gender and school type on these effects are analysed separately. The results reveal group differences of gender and school type for the unconditional growth model, while the conditional growth model highlights a moderating effect of school type on the relationship between legal norm acceptance and growth trajectories.

## Introduction

In social science or psychological research, it is often not only the analysis of linear effects that is of interest. Rather, multiplicative terms, such as moderation effects, are often the focus of research. According to Baron and Kenny (1986), a moderation effect can be defined as “a qualitative (e.g. sex, race, class) or quantitative (e.g. level of reward) variable that affects the direction and/or strength of the relation between an independent or predictor variable and a dependent or criterion variable” (Baron and Kenny, 1986, p. 1174). Once such a moderation effect is no longer to be investigated between two measured, manifest variables, but involves at least one unmeasured latent variable, it presents some methodological challenges. The analysis of moderating effects on latent growth factors in latent growth curve models (LGCMs) is one such case. In particular, the study of the moderating effects of categorical variables on unconditional and conditional LGCMs is of great interest in the social sciences. Once the focus of such an analysis moves from a linear to a curvilinear trend and/or focusses on non-normal distributed outcome variables, model building and estimation can become quite difficult. This paper therefore focusses on the problem of analysing categorical moderation effects in nonlinear LGCMs with nonnormal distributed outcome variables. One way to analyse the moderation effects between latent growth factors and (manifest) categorical variables is to add multiplicative interaction terms. As long as the categorical moderator variable is manifest, the estimation of such a term can be accomplished with relative ease. This can be achieved by incorporating the product term as an independent nominal scaled moderator variable within the model. In contrast, the integration of a latent interaction model is methodologically challenging, although some possibilities for integration have been discussed in the methodological literature in recent years. Examples are the so-called *product indicator approaches* (see for example Jöreskog & Wallentin, 1996; Kenny & Judd, 1984; Marsh et al., 2004), *Latent Moderated Structural Equations (LMS) approach* (Klein & Moosbrugger, 2000; Klein & Muthén, 2007) or Bayesian approaches (Lee, 2007; Asparouhov & Muthén, 2019; Kelava & Brandt, 2023).

Although it is generally possible to analyse the moderating effects of (manifest or latent) categorical variables by adding multiplicative interaction terms, the complexity of the model increases dramatically as the number of values of categorical variables and the number of latent growth factors increases. In combination with the high complexity of the methods themselves, the analyses with the aforementioned methods can be extremely cumbersome in practice.

The technique of multiple-group comparison is another possible and very obvious way to analyse the moderating effects of (categorical) variables within a LGCM (see for example Baron & Kenny, 1986; Jöreskog, 1971; Sörbom, 1974). In this method, individual models are estimated on a group-specific basis. By restricting the parameters across all groups, it is possible to analyse differences in the parameters between the groups, and thus any moderation by the grouping variable. The application of multiple-group comparisons is straightforward and can accommodate binary, ordinal, or count as dependent variables. Therefore, the flexibility of the method in its application, both in terms of distributional assumptions and modeling techniques, is very high. Furthermore, programs to model structural equations like M*plus* (Muthén & Muthén, 1998-2017) or *lavaan* (Rosseel, 2012) contain several features for multiple-group comparisons. However, when calculating models with latent variables and non-normally distributed outcome variables, it may be necessary to resort to computationally intensive numerical integration algorithms. This means that when using a classical maximum likelihood approach, it is often necessary to restrict ourselves to a few latent variables or random effects (Asparouhov & Muthén, 2021c; Muthén & Muthén, 1998-2017). This can lead to problems in computing multiple-group LGCMs with non-normally distributed data, particularly when there are a considerable number of latent growth factors and/or additional non-normally distributed (latent) variables present in the model.

This problem can be partially circumvented by analysing the models in a Bayesian framework using Markov Chain Monte Carlo (MCMC) techniques. Although the computational time for such Bayesian models is generally higher compared to classical maximum likelihood methods, this need not be the case for non-normally distributed data. In the Bayesian approach, the computational time increases only linearly, whereas with the maximum likelihood methods, the computational time increases exponentially with the number of latent variables (Asparouhov & Muthén, 2021c). Therefore, it is often practically impossible to estimate more complex models using classical maximum likelihood methods. However, Bayesian estimation is easily possible. Although Bayesian computation of such models for binary or ordinal data are implemented in various programs,^1^ a computation with count variables becomes recently possible, and even then, only in M*plus* (Asparouhov & Muthén, 2021c).^2^

In contrast to previous applications on this topic, we therefore apply a Bayesian approach to benefit from the advantages mentioned above. To demonstrate the capability of the Bayesian multiple-group approach for count distributed outcome variables, we use measurements of young people’s delinquent behaviour from the panel study ’Crime in the modern city’ (CrimoC) (Boers et al., 2010; Boers & Reinecke, 2019). This approach allows for a different interpretation of the estimated model parameters as conditional probabilities with respect to the parameters given by the data (see for example Depaoli, 2021; Kruschke, 2014). In addition to the advantages in terms of computational speed and model complexity, another advantage of the Bayesian approach is the ability to analyse small sample sizes that would cause problems in a classical frequentist treatment of latent growth curve models (Depaoli, 2021). Although our examples have a relatively large sample size, the Bayesian approach can easily be applied to data with a small number of cases. The following sections describe the basics of probability models for count outcomes and latent growth models, the inclusion of count variables in such models, their estimation in a Bayesian framework, and the extension to techniques of multiple-group comparisons. Subsequently, a detailed discussion of the data and models is provided, followed by the results of several separate multi-group analyses of unconditional and conditional growth models by gender and school type. The primary emphasis is on the easy implementation of the models utilising the M*plus* programme, with a subsequent focus on the straightforward presentation of results employing the R programming language.

## Probability models of count outcomes

### Poisson model

In the case of a dependent variable that represents the count of a particular event (count variable), the Poisson distribution is often used as a basis, which indicates the probability of the occurrence of an event in a given spatial or temporal frame:

1 $$\begin{aligned} Pr(Y=k) = \frac{e^{-\mu }\mu ^{k}}{k!} \end{aligned}$$

where *Y* is the random count response variable, *k* is the number of occurrences and $\mu$ is the so-called rate parameter, which is equal to the expected value *E*(*Y*). The Poisson regression is therefore

2 $$\begin{aligned} E(Y) = \mu = \exp (x'\beta ) \end{aligned}$$

where *x* represents the linear predictor variable(s) and $\beta$ is the vector of regression coefficients (Hilbe, 2011; Seddig, 2023; Reinecke, 2014, 2024). The Poisson distribution is characterised by the equidispersion constraint, i. e. $Var(Y)=E(Y)=\mu =\exp (x'\beta )$. If *Var*(*Y*) and *E*(*Y*) cannot be assumed to be equal, bias in parameter estimates and standard errors has to be expected.

### Negative binomial model

The negative binomial distribution is the alternative to the Poisson distribution in case the equidispersion does not hold. A so-called dispersion parameter for the negative binomial model can be specified in several ways. The so-called NB-2 parameterisation described in Hilbe (2011) is implemented in in M*plus* (Muthén & Muthén, 1998-2017).

By adding a gamma distributed heterogeneity parameter *u* with a mean of 1 and a variance of 1/*r*, the poisson probability distribution in Eq. 1 can be extended to a Poisson-gamma mixture distribution as follows:

3 $$\begin{aligned} Pr(Y=k) = \frac{e^{-\mu u}(\mu u)^{k}}{k!} \end{aligned}$$

Integrating this probability distribution according to *u* leads to the negative binomial distribution (see also Hilbe, 2011):

4 $$\begin{aligned} P(Y=k)=\frac{\Gamma (k+r)}{\Gamma (k+1)\Gamma (r)}p^{r}(1-p)^{k} \end{aligned}$$

where *Y* is again the random count response variable, *k* is the number of occurrences and *p* is a helper variable which is defined as $p = 1/(1+\delta \mu )$ with the rate parameter $\mu$ and the dispersion parameter $\delta$. $\Gamma$ simply denotes the gamma function and the variance of *Y* is given as $Var(Y)=\mu +\delta \mu ^{2}$. Finally, $\delta$ corresponds to 1/*r*. In particular, the definition of *Var*(*Y*) shows that the Poisson distribution is a special case of the negative binomial distribution if $\delta \rightarrow 0$. Accordingly, Eq. 2 also applies in case of a negative binomial distribution. The following explanations and examples refer to the negative binomial model because the distribution of the data used in the applications are not equidispersed.

## Latent growth curve model

Since the work of Tucker (1958) and Rao (1958), the general analysis of developmental trajectories using growth curve models has been of great interest for researchers in various disciplines. Following this initial work, Meredith and Tisak (1990) explored that growth models can be specified as a special case of confirmatory factor analysis within the general structural equation modeling framework. This is commonly referred to as *latent curve model* (Bollen & Curran, 2006; Grimm & McArdle, 2023) or as *latent growth curve model* (LGCM) (Hancock & Lawrence, 2006; Grimm et al., 2017). The latter one will be used here to refer to these models. The extension to count variables, (e.g., the incidence rate of delinquency) is relatively straightforward and has been applied by many authors in different research fields (for example Cambron et al., 2017; Friedman et al., 2009; Grimm & Stegmann, 2019; Seddig, 2023; Seddig & Reinecke, 2017). Due to the implementation of LGCMs in the structural equation modeling approach, it is also relatively straightforward to extend the models with group variables and to proceed multiple-group comparisons (Jöreskog, 1971; Sörbom, 1974). The *multiple-group latent growth curve model* (MG-LGCM) can be used as a moderation analysis of the particular categorical group variable (Baron & Kenny, 1986). Although the LGCM modeling described so far has been developed in a classical frequentist framework, an application in a Bayesian framework is easily possible. For a better understanding of the general model structure, the basics are briefly explained in the following two sections: the extension to count variables and categorical groups and the estimation in a Bayesian framework.

### Model equations

Suppose that we have a $t \times 1$ vector of (normally distributed) outcome variables $y_{t}$ with *t* time points. The relationship between the $m \times 1$ latent growth factor vector $\eta _{m}$ and the observed outcome variable can be described by the following equation (Meredith & Tisak, 1990, p. 108):

5 $$\begin{aligned} y_{t} = \tau _y + \Lambda _{y}\eta _{m}+\epsilon _{t} \end{aligned}$$

Where $\tau _y$ is the $t \times 1$ vector of the intercept of the manifest variable *y*,^3^$\Lambda _{y}$ is the $t \times m$ factor loading matrix and $\epsilon _{t}$ is the $t \times 1$ vector of the measurement errors. The latent growth curve factors $\eta _m$ can be further described as the sum of the $m \times 1$ vector of means of the latent growth curve factor $\alpha _m$ and the $m \times 1$ residual vector $\zeta _m$:

6 $$\begin{aligned} \eta _m = \alpha _m + \zeta _m \end{aligned}$$

If exogenous (latent or manifest) variables are considered in the model, Eq. 6 can be extended as follows:

7 $$\begin{aligned} \eta _m = \alpha _m + \Gamma \xi _{n} + \zeta _m \end{aligned}$$

where $\Gamma$ is the ($m \times n$) matrix of the regression coefficients of the ($n \times 1$) exogenous variables $\xi _n$.

Now, we assume that *y* is a count variable following a negative binomial distribution and that the probability function for *y* is given by Eq. 4. According to NB-2 parameterisation (Hilbe, 2011), the structural equation in Eq. 5 can now be integrated into the negative binomial distribution using Eq. 2 and the relationship $p = 1/(1+\delta \mu )$:

8 $$\begin{aligned} p = \frac{1}{1+\delta \exp (\Lambda _{y}\eta _{m})} \end{aligned}$$

We can now conclude that

9 $$\begin{aligned} E(y_{t})=\mu =\exp (\Lambda _{y}\eta _{m}) \end{aligned}$$

and accordingly

10 $$\begin{aligned} \ln E(y_{t})=\ln \mu =\Lambda _{y}\eta _{m} \end{aligned}$$

Thus, in the presence of a count outcome variable, the latent growth model corresponds to growth in the natural logarithm of $E(y_t)$.^4^ Here, $\delta$ denotes the so-called dispersion parameter, which is estimated instead of the residual variance $\epsilon$ in the case of a negative binomial distributed variable. Equation 6 applies as described (see also Asparouhov & Muthén, 2021c; Hilbe, 2011; Seddig, 2023).

The size of the factor loading matrix $\Lambda _y$ and the number of latent growth curve factors have to be specified depending on the assumed growth curve. It is important to note that the above model implies the growth of the natural logarithm of the expected value of $y_t$. Consequently, growth is not modelled directly for the count variable, but always for the logarithmic values. For example, if linear growth is assumed, a $t \times 2$ factor matrix $\Lambda _y$ is specified (Eq. 11). The first column refers to the intercept, the second column to the linear slope. All factor loadings are restricted (Bollen & Curran, 2006, p. 37). If curvilinear growth is assumed, a $t \times 3$ matrix $\Lambda _y$ for a quadratic growth model is specified (Eq. 12). In addition to the intercept and linear slope, a third column is added to specify the quadratic slope. The factor loadings of the third column are simply the square of the values of the second column (Bollen & Curran, 2006, p. 91)^5^:

11 $$\begin{aligned} \Lambda _y= &  \begin{pmatrix} 1 & 0 \\ 1 & 1 \\ 1 & 2 \\ \vdots & \vdots \\ 1 & t-1 \end{pmatrix}\end{aligned}$$

12 $$\begin{aligned} \Lambda _y= &  \begin{pmatrix} 1 & 0 & 0 \\ 1 & 1 & 1 \\ 1 & 2 & 4 \\ \vdots & \vdots & \vdots \\ 1 & t-1 & (t-1)^2 \end{pmatrix} \end{aligned}$$

Finally, the residual variances and covariances of the latent growth factors ($\Psi _{mm}$) are specified in matrix $\Psi$:

13 $$\begin{aligned} \Psi = \begin{pmatrix} \Psi _{11} & & \\ \Psi _{21} & \Psi _{22} & \\ \vdots & \vdots & \ddots & \\ \Psi _{m1} & \Psi _{m2} & \dots & \Psi _{mm} \\ \end{pmatrix} \end{aligned}$$

For detailed descriptions of different model specifications and examples, see e.g., Grimm and McArdle (2023); Reinecke (2024).

### Bayesian approach

Estimating an LGCM using a Bayesian approach does not change the structural or measurement part of the model. In a Bayesian LGCM approach, we are interested in finding the conditional probability of the unknown parameters given the observed data (the so-called posterior distribution). Therefore, we update our prior knowledge in the form of the prior probability of these parameters with information from the likelihood derived from the observed data. In mathematical terms,

14 $$\begin{aligned} \text {posterior}\propto \text {likelihood}\times \text {prior} \end{aligned}$$

The prior can be either *informative* and contains a lot of information, *weakly informative* with very little information or *diffuse* with no information at all. Frequently used and particularly favourable prior distributions for the individual parameter groups ($\alpha _{m}$, $\Psi$, $\delta$) of an LGCM under the assumption of negative binomial distributed outcome variables are the normal distribution (shortcut $\mathcal {N}$; for $\alpha _{m}$), the inverse gamma distribution (shortcut $\mathcal{I}\mathcal{G}$; for $\delta$ and $\Psi$) and the inverse Wishart distribution (shortcut $\mathcal{I}\mathcal{W}$; for $\Psi$).^6^ The likelihood corresponds to the probability of the data given the parameters (for a more detailed description see e.g. Depaoli, 2021; Depaoli et al., 2023; Kruschke, 2014; Lee, 2007; Muthén & Asparouhov, 2012).

Markov Chain Monte Carlo (MCMC) techniques are used to draw conditional samples from the posterior distribution (Depaoli, 2021; Asparouhov & Muthén, 2010). Options such as the calculation of the *posterior median*, *highest density interval* (*HDI*) or the *probability of direction* (*pd*) can be used to analyse the posterior distributions of each parameter (Kruschke, 2014; Makowski et al., 2019). Thereby, the *posterior median* is simply the median value of the drawn posterior distributions, the *HDI* “indicates which points of a distribution are most credible, and which cover most of the distribution” (Kruschke, 2014, p. 87) and the *pd* “can be interpreted as the probability that a parameter (described by its posterior distribution) is strictly positive or negative (whichever is the most probable)” (Makowski et al., 2019, p. 4).

Unfortunately, a general model test for count data is not yet available. However, the *Posterior Predictive P-Value* (PPP) is available for normally or ordinally distributed data (Asparouhov & Muthén, 2021b; Meng, 1994; Gelman et al., 2013). The PPP has an interval between 0 and 1. A value of $>0.05$ is considered as good and a value close to 0.5 is considered as ideal. To test the null hypothesis of a set of parameter restrictions in a Bayesian LGCM, a Bayesian Wald test as proposed by Asparouhov and Muthén (2021a) can be used. The test statistic of the Bayesian Wald test has an asymptotic chi-square distribution, and one can compute a *p*-value for the null hypothesis similar to the frequentist approach.^7^ While the Bayesian Wald Test proves the null hypothesis of a set of parameter restrictions, a significant *p*-value can be interpreted as rejecting the null hypothesis and thus rejecting the parameter restrictions.

**Fig. 1:**
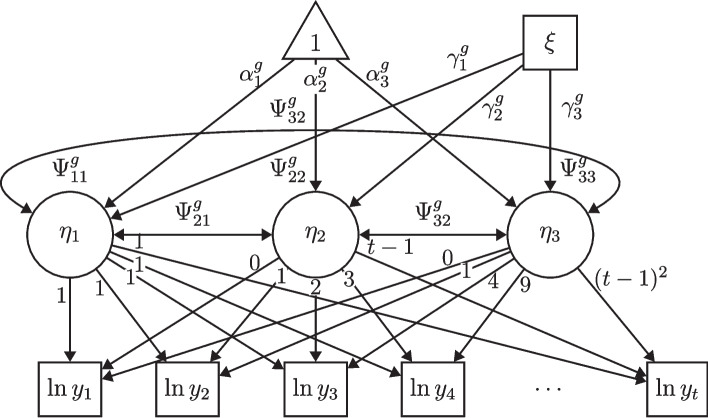
Conditional Multiple-Group Latent Growth Curve Model for Count Data (Quadratic Growth) *Note*: Dispersion parameter $\delta$ are not shown in the figure

### Moderation analysis with multiple-group modeling

#### Unconditional latent growth model

In the case of a categorical variable *g* with *G* categories, Eq. 5 is extended with superscript *g* which denotes group-specific vectors, matrices and variables (Bollen & Curran, 2006, p. 170):

15 $$\begin{aligned} y_t^g = \tau _y^g + \Lambda _y^g\eta _m + \epsilon _q^g \end{aligned}$$

$y^g$ represents a group-specific $t \times 1$ vector of outcome variables, $\Lambda _y^g$ denotes the group-specific factor loading matrix, $\eta _m$ denotes the latent growth factor vector and $\epsilon _q^g$ refers to the group-specific vector of measurement errors.^8^ The latent growth factors $\eta _m$ can be described as the sum of the group-specific means $\alpha _m^g$ and the group-specific residuals $\zeta _m^g$ (Bollen & Curran, 2006, p. 171):

16 $$\begin{aligned} \eta _m = \alpha _m^g + \zeta _m^g \end{aligned}$$

In the case of an unconditional growth curve model, the group-specific residual covariance matrix $\Psi ^g$ contains variances and covariances of the latent growth factors $\eta _m^g$. If the outcome variable is specified as a count, Eq. 8 is applied accordingly

17 $$\begin{aligned} p^g = \frac{1}{1+\delta ^g\exp (\Lambda _{y}^g\eta _{m})} \end{aligned}$$

As before, the measurement and structural parts remain the same in a Bayesian approach. However, the group-specific parameters listed above need not be estimated in all groups without restrictions. The multiple-group techniques allow restrictions of single parameters or complete vectors and matrices across groups to test for group invariances. The possibility of specifying specific restrictions also includes to test for which parameters a moderation of the categorical group variable *g* occurs. In case of the complete restriction of the factor loading matrix $\Lambda _y$ described above, only the effects of *g* on all other parameters can be investigated, but not the relationship between the latent growth factors $\eta _m$ and the outcome variable $y_t$. If some parameters of $\Lambda _y$ are freely estimated, the influence of *g* can be estimated. Only the latter one would be considered as a moderation of *g*. In the Bayesian framework, for example, a Bayesian Wald test as described above can be applied to test the restrictions of multiple parameters or entire groups of parameters.

#### Conditional latent growth model

The general formulae and explanations of the previous section also apply when one or more exogenous variables are added to the model. Only Eq. 16 has to be supplemented by the (group-specific) matrix $\Gamma ^g$ which contains the regression parameters of the (manifest or latent) exogenous variable(s) $\xi _n$, as in the general LGCM (Bollen & Curran, 2006, p. 172):

18 $$\begin{aligned} \eta _m = \alpha ^g_m + \Gamma ^g\xi _{n} + \zeta ^g_m \end{aligned}$$

Now matrix $\Psi$ is a residual (co)variance matrix. The formula indicates that it is possible to analyse the moderation of *g* on the influences of exogenous variables on the developmental trajectories (see also Fig. 1). As before, it is possible to test the moderating effect using a Bayesian Wald test.

## Empirical application: Juvenile delinquency

To further illustrate the use of a multiple-group comparison as an option for moderation analyses, several LGCMs are examined in the following section. Initially, group differences regarding an unconditional LGCM with respect to first gender and then school type are analysed separately. Subsequently, the moderation effects of gender and school type on conditional juvenile delinquency LGCMs are examined again separately. In the case of the conditional LGCM, the latent variable of legal norm acceptance is used as an exogenous variable. The variable of gender was chosen for analysis because it is one of the most widely recognized influences on juvenile delinquency. Explanations for this phenomenon range from differences in neurological development to social phenomena (Bennett et al., 2005; Boers & Reinecke, 2019; Painter & Farrington, 2004; Loeber et al., 2015). School type is included as a proxy for differences in educational attainment, which is also a well-known explanatory factor for juvenile delinquency (Maguin & Loeber, 1996; Hoffmann et al., 2013; Boers & Reinecke, 2019). The data are provided by the long-term panel study ’Crime in the modern city’ (CrimoC), which examines, the emergence and decline of delinquency in adolescence and young adulthood in the German city of Duisburg (Boers et al., 2010; Seddig & Reinecke, 2017; Boers & Reinecke, 2019). The study consists of a cohort of 7th graders in 2002 who were interviewed annually until 2009 and afterwards biannually until 2019. The average age of the participants in the first survey was 13 years. The outcome variable was constructed as the sum of the incidence (frequency) of 15 different self-reported delinquent behaviours: car theft, theft from cars, theft from vending machines, burglary, bicycle theft, shoplifting, other theft, fencing, assault with and without a weapon, bag snatching, robbery, spraying graffiti, scratching windows.

**Table 1:** Descriptive Statistics of Self-Reported Delinquency

	Delinquency							
	Age 13	Age 14	Age 15	Age 16	Age 17	Age 18	Age 19	Age 20
$\bar{x}$	2.53	5.36	5.14	3.88	3.21	1.80	1.29	0.58
sd	11.27	21.96	21.30	19.79	17.06	12.38	13.81	5.93
n	1,668	1,847	1,866	1,905	1,881	1,866	1,885	1,870

According to several empirical results of previous long-term studies (e.g., Cambridge Study, cf. Farrington et al. (2023); Dunedin Study, cf. Poulton et al. (2015)), a curvilinear development of delinquency during adolescence and young adulthood is expected. More or less, the peak of delinquency can be expected in the (early) teenage years (Loeber & Farrington, 2014). To estimate a curvilinear trajectory, an unconditional quadratic LGCM is specified as the base model (cf. Equation 12) using the first eight panel waves (13 to 20 years of age) of the CrimoC study. Regarding gender, it is expected that males will show a more extreme and steeper developmental curve than women (Painter & Farrington, 2004; Loeber et al., 2015). Regarding school type it is expected that the educational level of schools will be associated with a less extreme development of delinquency (Maguin & Loeber, 1996; Hoffmann et al., 2013). In our study four German school types are distinguished: *Gymnasium* (GYM), *Gesamtschule* (GES), *Realschule* (RE) and *Hauptschule* (HS). The *Gymnasium* is the school type with the highest educational degree in Germany, the *Realschule* offers a middle educational degree, and the *Hauptschule* has the lowest. The *Gesamtschule* is a mixture of the other three and offers all educational degrees.^9^ These schools start their educational program with students from the fifth grade. The Crimoc study started the survey with students from the seventh grade in 2002. The school type from the first panel wave is used as a grouping variable. Finally, the influence of the acceptance of legal norms in the first year of the survey on the development of delinquency and the moderation of this effect by gender and school type of the participants will be analysed. Theoretically, a negative effect of legal norm acceptance on the development of delinquency is expected (Seddig, 2014; Bentrup, 2014). Therefore, people with a higher initial acceptance of legal norms should have both a lower starting point and a flatter course of delinquency. To examine these relationships, we extend the basic unconditional LGCM to a conditional model. In a final step, the moderation of gender and school type is analysed in a multiple-group analysis. The acceptance of legal norms is introduced into the model as a latent variable measured by three items conducted in the first wave (t0722, t0725, t0727). The items are measured on a 5-point Likert scale. Each variable indicates agreement with statements about reasons for not committing a crime (1 = does not apply at all, 5 = fully applies, see Table A1 in the appendix for item wording). The measurement invariance of the latent variable is investigated to test the moderation of the conditional LGCM separately with respect to gender and school type, using distinct multiple-group LGCMs for each factor.

Overall, the database consists of a sample size of $n=1,945$. With regard to gender variable, this basic sample is divided into $n~=~822$ male and $n~=~1,123$ female participants. Due to missing data the sample is reduced to $n~=~1,851$ when analysing the effects of school type. Of these, $n~=~470$ are in the GYM group, $n~=~610$ in the GES group, $n~=~433$ in the RS group and $n~=~338$ in the HS group. Descriptive statistics of the outcome variable for each of the eight panel waves are given in Table 1. All models are estimated with the software M*plus*, Version 8.8 (Muthén & Muthén, 1998-2017) using the Bayesian framework and assuming a negative binomial distribution of the outcome variable.^10^ For each estimated model, 50,000 post-warm-up MCMC iterations are used on 4 parallel chains. Each model converged. As all models presented are Bayesian LGCMs, prior distributions for all parameter sets ($\Psi ^g$, $\delta$, $\alpha _{m}^g$; see also Eqs. 8 and 16) must be specified in advance. For simplicity, the default M*plus* options have been used for these examples: $\Psi ^g \sim \mathcal{I}\mathcal{W}(0,-4)$, $\delta \sim \mathcal{I}\mathcal{G}(-1,0)$, $\alpha _{m}^g \sim \mathcal {N}(0,\infty )$ and in case of the conditional LGCM additionally $\lambda ^g \sim \mathcal {N}(0,5)$ for the factor loadings of the latent variable (legal norms) and $\beta ^g \sim \mathcal {N}(0,\infty )$ for the regression effects. All prior distributions can be considered as *diffuse* and therefore do not contain prior information about the parameters. This means that no ‘direction’ or ‘magnitude’ of the parameters is preferred in the default prior distributions. In principle, they are almost flat probability distributions that assign the same probability to each possible value. One possible alternative would be to use more informative prior distributions, for example, assuming that the mean of the latent intercept is higher for men than for women. Nevertheless, in view of the large *n*, the question of prior distributions appears to be of little consequence, and the use of default options seems to be a suitable and straightforward option. Examples and explanations of the specification of more informative prior distributions can be found in the online Appendix.

### Effect of gender on the unconditional growth model

The first example is an examination of the effect of gender on the latent growth trajectories of delinquency using a multiple-group latent growth curve model for count data (MG-LGCM). The focus of interest is the effect of gender on the means, variances, and covariances of the latent growth factors, and thus on the developmental trajectories of delinquency.

**Fig. 2:**
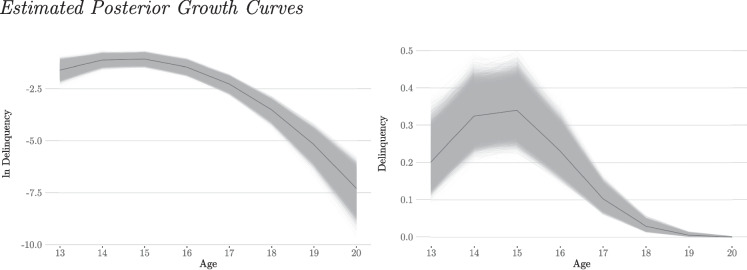
*Note*: The thick lines indicate the median posterior trajectory. The thin lines indicate all calculated posterior trajectories

At first, a base LGCM is constructed. In accordance with theoretical assumptions on the development of delinquency in adolescence and previous analyses with the CrimoC data, a quadratic course of delinquency is specified (cf. Boers & Reinecke, 2019; Seddig & Reinecke, 2017).^11^ The estimated growth curve of the base LGCM is shown in Fig. 2 for the delinquency rate (figure on the right) and the logarithmic delinquency rate (figure on the left).^12^ Both figures clearly show the curvilinear development of delinquency. This is particularly evident when using the logarithmic delinquency implied by the model in the left figure (see also Eq. 10). The exponentiated curves on a count scale show a flattening trend towards zero. However, this result is consistent with the count data models described in the previous sections. Therefore we can expect that delinquency rates will peak at the age of 15 and then decline.

**Fig. 3:**
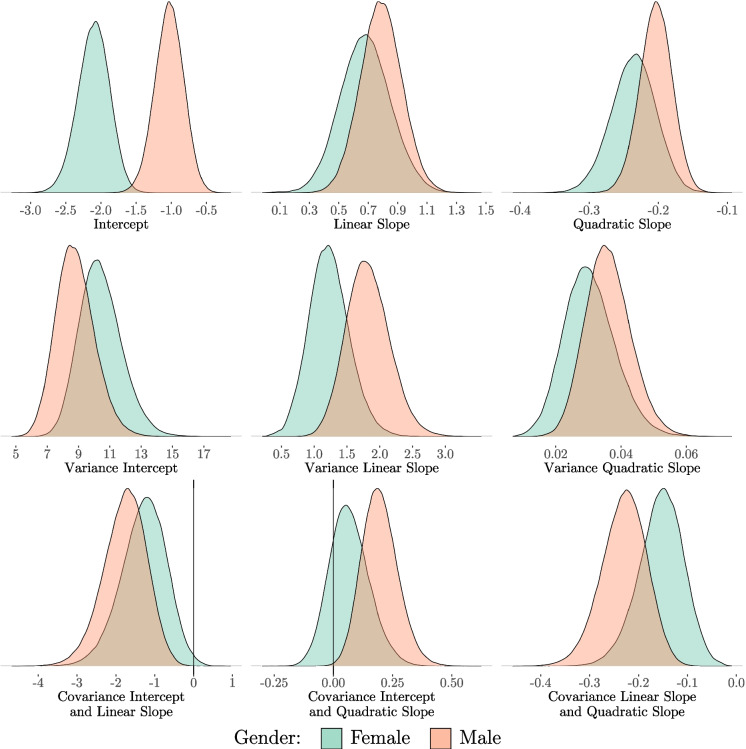
Posterior Distributions of the Latent Growth Factors (Gender/Unrestricted Model)

**Table 2:** Description of Posterior Distributions (Gender/Unrestricted Model)

	Male			Female		
	Median	95% HDI	pd	Median	95% HDI	pd
Mean (I)	$-$1.03	[$-$1.44, $-$0.64]	100.00%	$-$2.10	[$-$2.56, $-$1.68]	100.00%
Mean (S)	0.79	[0.51, 1.06]	100.00%	0.68	[0.35, 1.02]	100.00%
Mean (Q)	$-$0.20	[$-$0.25, $-$0.16]	100.00%	$-$0.24	[$-$0.30, $-$0.17]	100.00%
Variance (I)	8.74	[6.61, 11.42]	100.00%	10.33	[8.04, 13.33]	100.00%
Variance (S)	1.80	[1.22, 2.51]	100.00%	1.22	[0.66, 1.88]	100.00%
Variance (Q)	0.04	[0.02, 0.05]	100.00%	0.03	[0.02, 0.05]	100.00%
Covariance (I & S)	$-$1.74	[$-$2.95, $-$0.77]	99.99%	$-$1.25	[$-$2.52, $-$0.23]	99.25%
Covariance (I & Q)	0.19	[0.06, 0.36]	99.84%	0.06	[$-$0.08, 0.24]	78.85%
Covariance (S & Q)	$-$0.23	[$-$0.33, $-$0.15]	100.00%	$-$0.15	[$-$0.25, $-$0.07]	100.00%

*Note*: I = Intercept; S = Linear Slope; Q = Quadratic Slope; HDI = Highest Density Interval; pd = Probability of Direction; All values are on a logarithmic scale

To answer the question of how strong the estimated growth curves are influenced by gender, the base LGCM is extended to a MG-LGCM in which all means, variances, and covariances are unconstrained and freely estimated between the two groups (male, female). The posterior distributions of all freely estimated parameters are shown in Fig. 3. The corresponding statistics, such as the medians of the posterior distributions, the associated *HDI*s and the *pd*, can be found in Table 2.^13^

Looking at both the overall posterior distribution and the corresponding *HDI* and *pd*, it is noticeable that the intercept of the latent growth factor appears to be unequal between the two groups. The overlap between the two posterior distributions of the male and female groups is small, and the *HDI*s of the two groups also indicate different intercepts. In contrast, when looking at the posterior distribution of the quadratic slope, it is immediately apparent that the two groups are very similar. This first impression is reinforced when looking at the corresponding *HDI*s, with only minor differences between the upper and lower limits for males and females (e.g. 95% *HDI*s: male [$-$0.25, $-$0.16]; female [$-$0.30, $-$0.17]). Finally, the linear slope is a less clear-cut case. The posterior distributions overlap slightly and this is also evident when looking at the *HDI*s (e.g., 95% *HDI*s: male [0.51, 1.06]; female [0.35, 1.02]). It should also be noted that the variances and covariances are quite ambiguous. Therefore, a closer look is necessary.

One strategy is the Bayesian Wald test mentioned above. Additional parameter restrictions are added step-by-step to find the most parsimonious model that is not rejected (see Table A3 in the Appendix for detailed results). The first step is to test whether the null hypothesis of equality of covariances between both groups could be rejected. The results of the Wald test indicate that this is not the case. Therefore, the null hypothesis of the additional equality of the means of the quadratic slopes and the corresponding variances is tested in the next step. Again, the Wald test shows that this null hypothesis cannot be rejected either. Next, the equality of the linear slopes and the corresponding variances are also tested. However, these equality restrictions can be rejected. Therefore, we also tested whether the variances of the linear slopes are equal in the two groups. According to the Wald test, this equality restriction cannot be rejected. In the last step, the equality of the variances of the intercepts is tested, which also cannot be rejected. Therefore, the Wald tests indicate that only the means of the intercepts and the linear slopes differ between males and females.

**Table 3:** Description of Posterior Distributions (Gender/Restricted Model)

	Male			Female		
	Median	95% HDI	pd	Median	95% HDI	pd
Mean (I)	$-$1.13	[$-$1.51, $-$0.77]	100.00%	$-$1.93	[$-$2.30, $-$1.58]	100.00%
Mean (S)	0.85	[0.63, 1.08]	100.00%	0.57	[0.35, 0.80]	100.00%
Mean (Q)	$-$0.21	[$-$0.25, $-$0.18]	100.00%	$-$0.21	[$-$0.25, $-$0.18]	100.00%
Variance (I)	9.45	[7.78, 11.39]	100.00%	9.45	[7.78, 11.39]	100.00%
Variance (S)	1.47	[1.03, 1.94]	100.00%	1.47	[1.03, 1.94]	100.00%
Variance (Q)	0.03	[0.02, 0.04]	100.00%	0.03	[0.02, 0.04]	100.00%
Covariance (I & S)	$-$1.47	[$-$2.32, $-$0.73]	100.00%	$-$1.47	[$-$2.32, $-$0.73]	100.00%
Covariance (I & Q)	0.13	[0.03, 0.24]	99.38%	0.13	[0.03, 0.24]	99.38%
Covariance (S & Q)	$-$0.18	[$-$0.25, $-$0.12]	100.00%	$-$0.18	[$-$0.25, $-$0.12]	100.00%

*Note*: I = Intercept; S = Linear Slope; Q = Quadratic Slope; HDI = Highest Density Interval; pd = Probability of Direction; All values are on a logarithmic scale

**Fig. 4:**
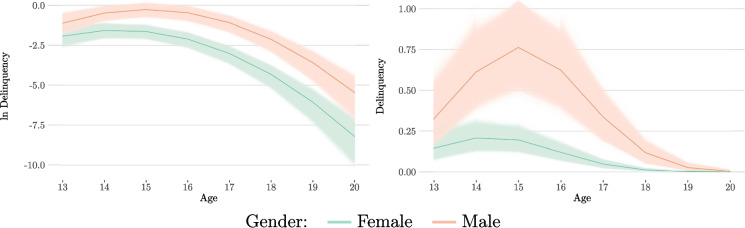
Estimated Posterior Growth Curves (Gender/Restricted Model) *Note*: The thick lines indicate the median posterior trajectory. The thin lines indicate all calculated posterior trajectories

In a final model, the quadratic slopes and (co)variances are equated, while both the intercepts and the linear slopes are freely estimated. Statistical measures for the posterior distributions of the means, variances and covariances of latent growth factors can be found in Table 3. Looking at both the posterior medians and the *HDI*s, we notice that the intercept is negative and that the male group has a higher value than the female group (male: $-$1.13; female: $-$1.93). This difference remains when looking at the *HDI*s (male [$-$1.51, $-$0.77]; female [$-$2.30, $-$1.58]). A similar pattern can be observed for the linear slopes, where the male group also has a higher posterior median than the female group (male: 0.85; female: 0.57). For both groups, a positive linear slope can be assumed with a very high degree of certainty (*pd*: 100%). However, there is an overlap in the *HDI*s (male [0.63, 1.08]; female [0.35, 0.80]). Therefore, a certain residual probability remains regarding the equality of the linear slopes in the two groups. Finally, the common quadratic slope is negative (posterior median: $-$0.21, *pd*: 100%). The corresponding posterior distributions of the growth curves for the two groups can be seen in Fig. 4. The figure on the left shows the model-implied expected values on a logarithmic scale. The figure on the right shows the same expected values on a count scale.

The results allow the conclusion that gender affects both the starting point of the development of delinquency (intercept) and the initial increase (linear slope). However, it does not seem to affect the flattening of the curve afterward (quadratic slope) or the variances and covariances of the latent growth factors. Boys start with a higher level of delinquency at the age of 13 and then show a stronger increase than girls. However, the variance of growth factors, the correlations between them, and the expected decline in delinquency in late adolescence are not affected by gender.

### Effect of school type on the unconditional growth model

The second example is an analysis of the effect of different school types on the growth trajectories of delinquency. Again, a quadratic growth model is assumed, and the effect on the means of the latent growth factors is of primary interest. Once again, the unconditional LGCM described in the previous section serves as the basis (see Table A2 and Fig. 2).

**Fig. 5:**
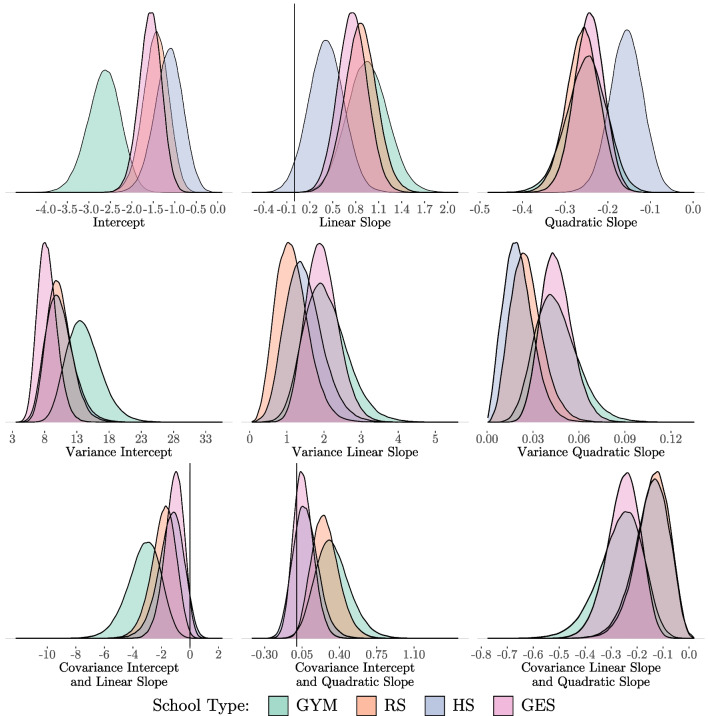
Posterior Distributions of the Latent Growth Factors (School Type/Unrestricted Model)

In a first step, a count MG-LGCM is estimated with all group-specific parameters released in all groups. The posterior distributions of the means, variances and covariances of the latent growth variables for the four groups are presented in Fig. 5. The corresponding summary statistics of the posterior distributions can be found in Table 4. In particular, when looking at the entire posterior distribution, it is noticeable that there is a large overlap between all school types in the linear and quadratic slopes, as well as in the variances and covariances. Even when looking at the corresponding *HDI*s, the parameters do not seem to differ much between the groups. However, the picture is slightly different for the intercepts. The posterior distributions of the RS group and the GES group clearly overlap to a large extent. The intercept for the GYM group appears to be much lower. The intercept for the HS group is slightly higher than the intercept of the RS group and GES group (e.g., 95% *HDI*s: GYM [$-$3.45, $-$1.93], RS [$-$2.05, $-$0.89], HS [$-$1.82, $-$0.52], GES [$-$2.10, $-$1.08]).

Using a stepwise procedure and the Bayesian Wald test, various parameter restrictions are tested to reach the most parsimonious model (see Table A4 in the Appendix for detailed results). The first step is a test of whether the null hypothesis of equality of covariances of the latent growth factors can be rejected. The results of the Wald test indicate that this is not the case. In a second step, the additional equality constraint of all growth factor variances is tested. Again, the null hypothesis cannot be rejected. Due to the high similarity of the linear and quadratic slopes in the groups, the equality constraint is proved together in a third step. The Wald test shows that the null hypothesis cannot be rejected either. In a fourth step, the parameter equality constraint of all intercepts is tested. This null hypothesis is rejected. Finally the intercept of the GYM group is released. Again, the null hypothesis could not be rejected.

A final MG-LGCM is estimated in which the corresponding parameters are equated. Some statistical measures describing the corresponding posterior distributions are given in Table 5. The linear slope equated for all groups is very likely and has a positive value (posterior median: 0.71, *pd*: 100%). The quadratic slope equated for all groups is also very likely and has a negative value (posterior median: $-$0.22, *pd*: 100%). The intercept of the GYM group has a lower posterior median value (posterior median: $-$2.21) than the restricted intercept of the RS/HS/GES group (posterior median: $-$1.44). The corresponding *HDI*s show that there is a certain probability that the intercept of the GYM group is lower than the intercept of the other three groups (e.g., 95% *HDI* GYM [$-$2.65, $-$1.79]; 95% *HDI* RS/HS/GES [$-$1.77, $-$1.12]).

**Fig. 6:**
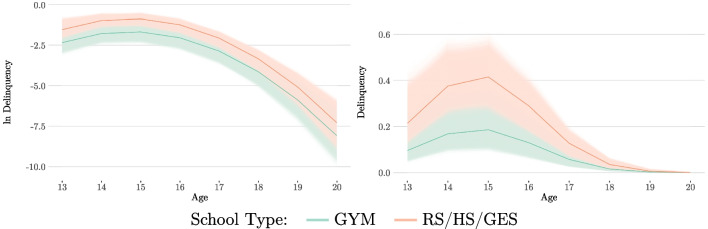
Estimated Posterior Growth Curves (School Type/Restricted Model) *Note*: The thick lines indicate the median posterior trajectory. The thin lines indicate all calculated posterior trajectories

In general, it can be concluded that the school type GYM school has an effect on the initial value (intercept) of the development of delinquency. However, no influence of school types can be observed on the subsequent development of delinquency (linear and quadratic slopes), which apparently becomes evident by inspection of the posterior growth trajectories. Although the starting point of the delinquency trajectories differs, the subsequent trajectories are not different and run parallel to each other in the logarithmic variant of the estimated posterior growth curves (left graph in Fig. 6).

### Moderation effect of gender and school type on the conditional growth model

In the next example, the moderation of gender and school type is estimated on a conditional LGCM. This model contains measurements of legal norm acceptance obtained from the first wave of the CrimoC study. Prior to the moderation analysis, a Bayesian confirmatory factor model is estimated to test the construct validity of the latent variable *legal norm acceptance*. Later, this model is used to analyse measurement invariance with respect to gender and school type. As described in detail in a previous section, acceptance of legal norms is measured by the three items (see also Table A1 in the appendix). A first test of the confirmatory factor model treating the variables as ordinal results in a very good model fit ($PPP~=~0.491$).^14^ To use the latent variable in a multigroup analysis, the (metric) measurement invariance of the factor has to be tested (Brown, 2015; Reinecke, 2014; Widaman & Olivera-Aguilar, 2023). Therefore, a baseline and a metric model of measurement invariance are estimated with group variables gender and school type. In the baseline model, no group restrictions are applied, whereas in the metric model, at least the factor loadings are set equal across groups. The baseline models for both group comparisons show the expected good model fit (gender: $PPP~=~0.496$; school type: $PPP~=~0.321$). The two metric models have an overall poorer, but still good model fit (gender: $PPP~=~0.435$; school type: $PPP~=~0.280$). Consequently, metric invariance of the factor model can be assumed across the categories of gender and school type.

**Table 4:** Description of Posterior Distributions (School Type/Unrestricted Model)

	GYM			RS			HS			GES		
	Median	95% HDI	pd	Median	95% HDI	pd	Median	95% HDI	pd	Median	95% HDI	pd
Mean (I)	$-$2.64	[$-$3.45, $-$1.93]	100.00%	$-$1.43	[$-$2.05, $-$0.89]	100.00%	$-$1.13	[$-$1.82, $-$0.52]	100.00%	$-$1.57	[$-$2.10, $-$1.08]	100.00%
Mean (S)	0.95	[0.44, 1.49]	99.99%	0.86	[0.45, 1.29]	100.00%	0.41	[$-$0.04, 0.86]	96.38%	0.76	[0.39, 1.14]	100.00%
Mean (Q)	$-$0.25	[$-$0.35, $-$0.16]	100.00%	$-$0.26	[$-$0.34, $-$0.18]	100.00%	$-$0.16	[$-$0.24, $-$0.08]	100.00%	$-$0.24	[$-$0.32, $-$0.18]	100.00%
Var. (I)	13.91	[9.52, 20.11]	100.00%	10.15	[6.98, 14.65]	100.00%	10.16	[6.68, 15.31]	100.00%	8.33	[5.83, 11.72]	100.00%
Var. (S)	2.00	[1.06, 3.35]	100.00%	1.11	[0.41, 2.11]	100.00%	1.42	[0.61, 2.58]	100.00%	1.93	[1.17, 2.89]	100.00%
Var. (Q)	0.04	[0.02, 0.08]	100.00%	0.03	[0.01, 0.05]	100.00%	0.02	[0.00, 0.04]	100.00%	0.04	[0.03, 0.07]	100.00%
CV (I & S)	$-$3.16	[$-$5.81, $-$1.20]	99.98%	$-$1.81	[$-$3.79, $-$0.38]	99.60%	$-$1.29	[$-$3.38, 0.23]	94.74%	$-$1.07	[$-$2.58, 0.08]	96.47%
CV (I & Q)	0.33	[0.05, 0.70]	99.22%	0.26	[0.05, 0.56]	99.39%	0.08	[$-$0.12, 0.35]	76.28%	0.06	[$-$0.10, 0.27]	74.53%
CV (S & Q)	$-$0.26	[$-$0.46, $-$0.12]	100.00%	$-$0.14	[$-$0.28, $-$0.03]	99.90%	$-$0.14	[$-$0.30, $-$0.03]	99.78%	$-$0.25	[$-$0.39, $-$0.14]	100.00%

*Note*: I = Intercept; S = Linear Slope; Q = Quadratic Slope; HDI = Highest Density Interval; pd = Probability of Direction; CV = Covariance; All values are on a logarithmic scale

**Table 5:** Description of Posterior Distributions (School Type/Restricted Model)

	GYM			RS/HS/GES		
	Median	95% HDI	pd	Median	95% HDI	pd
Mean (I)	$-$2.21	[$-$2.65, $-$1.79]	100.00%	$-$1.44	[$-$1.77, $-$1.12]	100.00%
Mean (S)	0.71	[0.48, 0.94]	100.00%	0.71	[0.48, 0.94]	100.00%
Mean (Q)	$-$0.22	[$-$0.26, $-$0.18]	100.00%	$-$0.22	[$-$0.26, $-$0.18]	100.00%
Variance (I)	9.70	[7.97, 11.71]	100.00%	9.70	[7.97, 11.71]	100.00%
Variance (S)	1.44	[1.03, 1.93]	100.00%	1.44	[1.03, 1.93]	100.00%
Variance (Q)	0.03	[0.02, 0.04]	100.00%	0.03	[0.02, 0.04]	100.00%
Covariance (I & S)	$-$1.48	[$-$2.36, $-$0.72]	100.00%	$-$1.48	[$-$2.36, $-$0.72]	100.00%
Covariance (I & Q)	0.14	[0.03, 0.26]	99.60%	0.14	[0.03, 0.26]	99.60%
Covariance (S & Q)	$-$0.18	[$-$0.25, $-$0.12]	100.00%	$-$0.18	[$-$0.25, $-$0.12]	100.00%

*Note*: I = Intercept; S = Linear Slope; Q = Quadratic Slope; HDI = Highest Density Interval; pd = Probability of Direction; The RS, HS and GES groups are reported as RS/HS/GES because all parameters are equated between these groups. All values are on a logarithmic scale

In the next step, the basic conditional LGCM is estimated and analysed for the whole sample. The posterior distributions of the regression paths of the latent variable norm acceptance on the latent growth factors are given in Table 6. The scale of each measurement of the norm variable indicates that the higher the scale value, the higher the norm acceptance. The median of the regression coefficients can be interpreted as follows: The higher the initial norm acceptance, the lower the starting point for the development of delinquency ($-1.39$). The higher the initial norm acceptance, the larger the subsequent increase in delinquency (0.29). The higher the initial norm acceptance, the larger the subsequent decline of delinquency ($-0.03$).

**Fig. 7:**
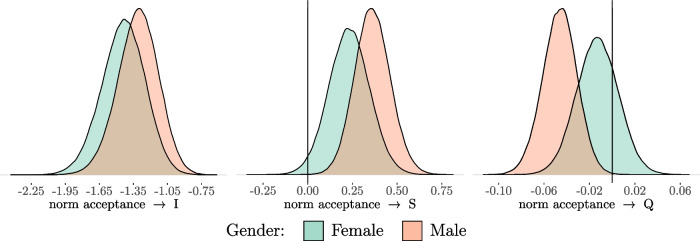
Posterior Distributions of the Regression Parameters (Gender) *Note*: I = Intercept; S = Linear Slope; Q = Quadratic Slope

Now the moderation of gender and school type can be explored in a further step. At first, we look in more detail at the result of a multiple-group model of the conditional LGCM with gender as a grouping variable. The regression parameters in each group are not restricted across both gender categories. The corresponding posterior distributions of the regression effects for both groups are given in Fig. 7 and their descriptions in Table 7. Obviously, the direction of the effects is the same for both groups. However, in some cases the probability of this direction is lower for females compared to males. For example, the *pd* of the regression effect of norm acceptance on the quadratic growth factor is only 76.04 % and therefore not very reliable. Finally, to test whether there are indeed differences in the regression effects between the groups, the Bayesian Wald test is used again (see Table A5 in the Appendix for detailed results). At first, the equality constraint of the regression coefficients of norm acceptance on the intercept of the LGCM is tested. The Wald test shows that this constraint cannot be rejected. Furthermore, the additional equality constraint of the regression coefficients of norm acceptance on the linear slopes of the LGCM cannot be rejected. The same applies for the equality constraint of the regression coefficients on the quadratic slopes. Thus, there is no difference of the regression coefficients with respect to gender which leads to the conclusion that gender does not moderate the regression effects of norm acceptance on the latent growth factors.

**Fig. 8:**
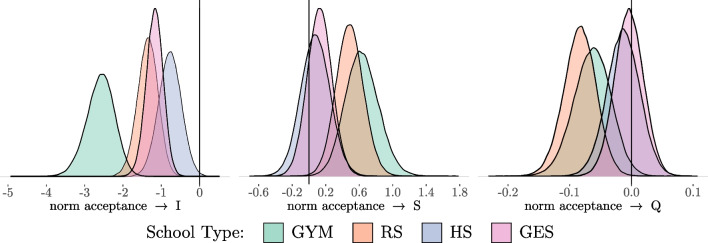
Posterior Distributions of the Regression Parameters (School Type) *Note*: I = Intercept; S = Linear Slope; Q = Quadratic Slope

The second model explores the moderation of school type on the conditional LGCM which still serves as the basic model (see the description in Table 6). As before, the first version of the conditional MG-LGCM contains no restrictions on the regression effects between each category of school type. The results of the posterior distributions of the regression effects in each group is shown in Fig. 8 and their descriptions in Table 8. In general, the direction of the effects does not seem to differ much between the school types. Differences between the direction of the parameters are small. Only the effect of norm acceptance on the intercept has a larger negative value for the GYM group ($-2.57$) than in the other three groups ($-0.77$, $-1.17$, $-1.35$).

**Table 6:** Description of Posterior Distributions (Conditional LGCM; Regression Parameters)

	Median	95% HDI	pd
norm acceptance $\rightarrow$ I	$-$1.39	[$-$1.68, $-$1.13]	100.00%
norm acceptance $\rightarrow$ S	0.29	[0.14, 0.45]	100.00%
norm acceptance $\rightarrow$ Q	$-$0.03	[$-$0.06, $-$0.01]	99.80%

*Note*: I = Intercept; S = Linear Slope; Q = Quadratic Slope; HDI = Highest Density Interval; pd = Probability of Direction; All values are on a logarithmic scale

**Table 7:** Description of Posterior Distributions (Conditional LGCM; Regression Parameters; Gender)

	Male			Female		
	Median	95% HDI	pd	Median	95% HDI	pd
norm acceptance $\rightarrow$ I	$-$1.31	[$-$1.68, $-$0.97]	100.00%	$-$1.44	[$-$1.83, $-$1.08]	100.00%
norm acceptance $\rightarrow$ S	0.36	[0.17, 0.56]	99.99%	0.23	[0.01, 0.46]	97.93%
norm acceptance $\rightarrow$ Q	$-$0.05	[$-$0.08, $-$0.02]	99.90%	$-$0.01	[$-$0.05, 0.02]	76.04%

*Note*: I = Intercept; S = Linear Slope; Q = Quadratic Slope; HDI = Highest Density Interval; pd = Probability of Direction; All values are on a logarithmic scale

**Table 8:** Description of Posterior Distributions (Conditional LGCM; Regression Parameters; School Type)

	GYM			RS		
	Median	95% HDI	pd	Median	95% HDI	pd
norm acceptance $\rightarrow$ I	$-$2.57	[$-$3.35, $-$1.91]	100.00%	$-$1.35	[$-$1.90, $-$0.84]	100.00%
norm acceptance $\rightarrow$ S	0.63	[0.26, 1.04]	99.96%	0.49	[0.17, 0.81]	99.88%
norm acceptance $\rightarrow$ Q	$-$0.06	[$-$0.12, $-$0.01]	98.56%	$-$0.08	[$-$0.14, $-$0.03]	99.93%
	HS			GES		
	Median	95% HDI	pd	Median	95% HDI	pd
norm acceptance $\rightarrow$ I	$-$0.77	[$-$1.37, $-$0.20]	99.54%	$-$1.17	[$-$1.62, $-$0.75]	100.00%
norm acceptance $\rightarrow$ S	0.08	[$-$0.26, 0.42]	67.12%	0.13	[$-$0.16, 0.42]	80.64%
norm acceptance $\rightarrow$ Q	$-$0.01	[$-$0.07, 0.04]	69.05%	0.004	[$-$0.05, 0.04]	57.19%

*Note*: I = Intercept; S = Linear Slope; Q = Quadratic Slope; HDI = Highest Density Interval; pd = Probability of Direction; All values are on a logarithmic scale

**Table 9:** Description of Posterior Distributions (Conditional LGCM; Regression Parameters; School Type; Partly Restricted Model)

	GYM			RS/HS/GES		
	Median	95% HDI	pd	Median	95% HDI	pd
norm acceptance $\rightarrow$ I	$-$2.01	[$-$2.55, $-$1.53]	100.00%	$-$1.20	[$-$1.50, $-$0.93]	100.00%
norm acceptance $\rightarrow$ S	0.30	[ 0.15, 0.46]	99.99%	0.30	[ 0.15, 0.46]	99.99%
norm acceptance $\rightarrow$ Q	$-$0.04	[$-$0.06, $-$0.01]	99.83%	$-$0.04	[$-$0.06, $-$0.01]	99.83%

*Note*: I = Intercept; S = Linear Slope; Q = Quadratic Slope; HDI = Highest Density Interval; pd = Probability of Direction; The RS, HS and GES groups are reported as RS/HS/GES because all parameters are equated between these groups. All values are on a logarithmic scale

The Bayesian Wald test is again applied to test whether this difference can be assumed as a moderation of school type (see Table A6 in the Appendix for detailed results). At first, it is tested whether a moderation of school type on the regression effect of norm acceptance on the latent growth factors could be generally excluded. The test shows that the null hypothesis of equality of the regression parameters in all groups is rejected. Accordingly, it can be assumed that the type of school serves as a moderator. For a more detailed analysis, the exclusion of the equality constraint of all regression parameters between the groups is tested (with the exception of the effect of norm acceptance on the intercept in the GYM group). This exclusion is also rejected. Therefore, it can be assumed that a moderation of school type on the influence of legal norm acceptance exists, but it is limited to the intercept for the GYM group.

A partly restricted model contains equality restrictions for the regression effects of norm acceptance on the intercept regarding three school types (RS/HS/GES) and equality restrictions for the regression effects of norm acceptance on the slope as well as on the quadratic slope regarding all school types. Looking at the results of the partly restricted model in Table 9 it can be observed that the regression parameter on the intercept has a higher negative value for the GYM group ($-2.01$). This result leads to the following substantive interpretations: The influence of young people’s acceptance of legal norms on the starting point for the development of delinquency is negative for all school types, i.e., a higher acceptance of legal norms corresponds to a lower initial level of delinquency, but an equally high acceptance of legal norms in the GYM group leads to an even lower initial level of delinquency than in the other types of school. With regard to the impact of norm acceptance on the subsequent development of delinquency, no differences are observed between the various school types. The previously observed general positive effect of norm acceptance on the initial increase can be observed in all school types, e.g. young people with a higher initial norm acceptance also show a greater increase in delinquency. The same can be concluded regarding the negative effect of norm acceptance on the quadratic growth factor. Regardless of school type, young people with higher initial norm acceptance have a greater decline of delinquency.

## Discussion

The paper provides a tutorial on the handling of categorical (moderator) variables in complex growth curve models with count outcome variables using multiple-group analyses (MG-LGCM). The objective of this paper is to demonstrate the application of Bayesian estimation techniques and illustrate the simplicity, clarity, and effective visualisation of the results with the software M*plus* and R, which could be achieved successfully.

All analyses are based on data from eight panel waves from the CrimoC study (Boers et al., 2010; Boers & Reinecke, 2019). Moderation effects of gender and school type on juvenile delinquency are examined using unconditional and conditional LGCMs for count data. Overall, moderation effects are found in all model variants described except for the moderation of gender on the relationship between legal norm acceptance and the development of delinquency. Furthermore, the use of a Bayesian MG-LGCM is an easily implemented alternative to other techniques of moderation analysis (e.g., the implementation of latent interaction variables). Although the LGCM in this tutorial is based on count-distributed outcome variables, other non-normally distributed variables can be considered. The extension of the MG-LGCM to other complex modeling strategies within the structural equation approach is straightforward, e.g., the *Parallel Process Growth Model* (Duncan et al., 2006; Wickrama et al., 2022), the *Random Intercept Crossed-Lagged Panel Model* (Hamaker et al., 2015; Mulder & Hamaker, 2021), the *Autoregressive Latent Trajectory Model* (Bollen & Curran, 2006) or the *Latent Curve Model with Structured Residuals* (Curran et al., 2014). Combining moderation and mediation effects can also be incorporated into these models.

However, the technique of multiple-group comparisons to analyse moderation effects has some limitations. The higher the number of categories of the moderation variable, the more groups have to be analysed simultaneously. With an increasing number of groups, the number of model variants with different parameter restrictions becomes increasingly large. One possible solution could be a type of mixture MG-LGCM in which groups with the same parameter are clustered into latent classes. Recently, de Roover (2021) proposed a mixture model for the analysis of measurement invariances in confirmatory factor models between large numbers of groups (see also Perez Alonso et al., 2023). However, a more detailed investigation of mixture modeling with regard to multiple-group analyses is still pending.

An increasing number of groups also increases the probability of small group sizes. Due to the potentially very small sample sizes for some groups, the prior distributions have a large influence on the parameter estimates. Therefore, prior sensitivity analysis is essential in empirical applications. Similarly, the use of more informative prior distributions could be beneficial and should be considered in further applications.

The models presented and discussed here are also limited to controlling for (manifest) categorical variables. Theoretically, the LGCM can be expanded to a growth mixture model to obtain different latent classes of trajectories (see e.g. Kreuter & Muthén, 2008). For example, the latent classes containing different delinquency trajectories obtained from the CrimoC data (Boers et al., 2014; Boers & Reinecke, 2019; Seddig & Reinecke, 2017) could be used to analyse a moderation of these classes on the influence of legal norm acceptance on the variables of the LGCM. However, this is beyond the scope of the paper.

In addition, there is the possibility of estimating moderations on growth models using multiple-group techniques, which has not yet been discussed. In the current models, the factor loadings of the LGCM have all been pre-specified to reflect specific growth. As a result, the influence of the growth factors on the delinquency variables is the same for all groups. Alternatively, it is possible to freely estimate some of the factor loadings (e.g., for an initial LGCM see McArdle & Epstein, McArdle and Epstein ; Meredith & Tisak, Meredith and Tisak ) and thus obtain group differences of the factor loadings and hence the developmental character of growth. However, this is also beyond the scope of the paper.

Furthermore, testing informative hypotheses (such as the equality of some parameters in the examples described) using the so-called Bayes factor (Kass & Raftery, 1995; Jeffreys, 1967; van de Schoot et al., 2012; Gu et al., 2019) can be of great benefit. Especially, in contrast to the Bayesian Wald test, it would also be possible to compare individual hypotheses in a more Bayesian sense using the Bayes factor. Different alternative hypotheses could be weighed against each other and evaluated according to their plausibility (see Kass and Raftery (1995) and van de Schoot et al. (2012) for more information on the Bayes factor).

## Conclusion

Although analysing moderation effects in complex statistical models can be challenging, there are effective methods available. One such method is the use of multiple-group comparisons within the structural equation modeling framework, which is especially useful when working with categorical group variables. This tutorial demonstrated the application of multi-group models with count outcome variables in a Bayesian context, focussing on group differences and moderation effects in relation to adolescent delinquency trajectories. The analysis started with an exploration of gender differences, followed by an examination of differences between school types. Finally, the tutorial addressed the moderation effects of these variables in the conditional growth model, with legal norm acceptance as an exogenous variable. In conclusion, this approach provides an effective and easily manageable way to conduct multiple-group analysis and especially moderation analysis of categorical variables, particularly when the outcome variables are count data.

## Data Availability

The dataset used for the examples shown is available at the Open Science Framework: https://osf.io/9jvn7/

## Appendix

**Table 10:** Item Wording of Legal Norm Acceptance

	It is better not to commit crimes because...
t0722	it is important to respect the law
t0725	one harms others who cannot help it
t0727	I harm myself in the process

**Table 11:** Description of Posterior Distributions

	Median	95% HDI	pd
Mean (I)	$-$1.60	[$-$1.92, $-$1.29]	100.00%
Mean (S)	0.69	[0.47, 0.92]	100.00%
Mean (Q)	$-$0.22	[$-$0.26, $-$0.18]	100.00%
Variance (I)	9.61	[7.95, 11.56]	100.00%
Variance (S)	1.51	[1.09, 1.99]	100.00%
Variance (Q)	0.03	[0.02, 0.04]	100.00%
Covariance (I & S)	$-$1.44	[$-$2.29, $-$0.71]	100.00%
Covariance (I & Q)	0.13	[0.03, 0.25]	99.52%
Covariance (S & Q)	$-$0.19	[$-$0.25, $-$0.13]	100.00%

*Note*: I = Intercept; S = Linear Slope; Q = Quadratic Slope; HDI = Highest Density Interval; pd = Probability of Direction; All values are on a logarithmic scale

**Table 12:** Results Bayesian Wald Test (Gender/Unconditional Growth Model)

Hypothesis		$\chi ^2$	*df*	*p*
Equality of Covariances	$COV^{male} = COV^{female}$	3.539	3	.316
Additional equality of Mean (Q) and Variance (Q):	$COV^{male} = COV^{female}$			
	$$\alpha _{Q}^{male} = \alpha _{Q}^{female}$$			
	$\zeta _{Q}^{male} = \zeta _{Q}^{female}$	9.261	5	.099
Additional equality of Mean (S) and Variance (S):	$COV^{male} = COV^{female}$			
	$$\alpha _{S,Q}^{male} = \alpha _{S,Q}^{female}$$			
	$\zeta _{S,Q}^{male} = \zeta _{S,Q}^{female}$	40.568	7	$<.001$
Additional equality of Variance (S):	$COV^{male} = COV^{female}$			
	$$\alpha _{Q}^{male} = \alpha _{Q}^{female}$$			
	$\zeta _{S,Q}^{male} = \zeta _{S,Q}^{female}$	9.281	6	.158
Additional equality of Variance (I):	$COV^{male} = COV^{female}$			
	$$\alpha _{Q}^{male} = \alpha _{Q}^{female}$$			
	$\zeta _{I,S,Q}^{male} = \zeta _{I,S,Q}^{female}$	10.894	7	.143

*Note*: I = Intercept; S = Linear Slope; Q = Quadratic Slope; *COV* = Covariance; $\alpha$ = Mean(s) of Latent Growth Factor(s); $\zeta$ = Variance(s) of Latent Growth Factor(s)

**Table 13:** Results Bayesian Wald Test (School Type/Unconditional Growth Model)

Hypothesis		$\chi ^2$	*df*	*p*
Equality of Covariances	$COV^{GYM} = COV^{RS} = COV^{HS} = COV^{GES}$	14.11	9	.118
Additional equality of Variance (I,S,Q):	$COV^{GYM} = COV^{RS} = COV^{HS} = COV^{GES}$			
	$\zeta _{I,S,Q}^{GYM} = \zeta _{I,S,Q}^{RS} = \zeta _{I,S,Q}^{HS} = \zeta _{I,S,Q}^{GES}$	22.66	18	.204
Additional equality of Means (S,Q):	$COV^{GYM} = COV^{RS} = COV^{HS} = COV^{GES}$			
	$$\zeta _{I,S,Q}^{GYM} = \zeta _{I,S,Q}^{RS} = \zeta _{I,S,Q}^{HS} = \zeta _{I,S,Q}^{GES}$$			
	$\alpha _{S,Q}^{GYM} = \alpha _{S,Q}^{RS}= \alpha _{S,Q}^{HS}= \alpha _{S,Q}^{GES}$	27.95	24	.262
Additional equality of Means (I):	$COV^{GYM} = COV^{RS} = COV^{HS} = COV^{GES}$			
	$$\zeta _{I,S,Q}^{GYM} = \zeta _{I,S,Q}^{RS} = \zeta _{I,S,Q}^{HS} = \zeta _{I,S,Q}^{GES}$$			
	$\alpha _{I,S,Q}^{GYM} = \alpha _{I,S,Q}^{RS}= \alpha _{I,S,Q}^{HS}= \alpha _{I,S,Q}^{GES}$	47.88	27	$<.01$
Additional release of Mean (I) of the GYM group:	$COV^{GYM} = COV^{RS} = COV^{HS} = COV^{GES}$			
	$$\zeta _{I,S,Q}^{GYM} = \zeta _{I,S,Q}^{RS} = \zeta _{I,S,Q}^{HS} = \zeta _{I,S,Q}^{GES}$$			
	$\alpha _{I,S,Q}^{RS}= \alpha _{I,S,Q}^{HS}= \alpha _{I,S,Q}^{GES}$	30.32	26	.255

*Note*: I = Intercept; S = Linear Slope; Q = Quadratic Slope; *COV* = Covariance; $\alpha$ = Mean(s) of Latent Growth Factor(s); $\zeta$ = Variance(s) of Latent Growth Factor(s)

**Table 14:** Results Bayesian Wald Test (Gender/Conditional Growth Model)

Hypothesis		$\chi ^2$	*df*	*p*
Equality of regression coefficients on the Intercept:	$\beta _1^{male} = \beta _1^{female}$	0.283	1	.595
Additional equality of regression coefficients on the Linear Slope:	$\beta _1^{male} = \beta _1^{female}$ $\beta _2^{male} = \beta _2^{female}$	2.543	2	.282
Additional equality of regression coefficients on the Quadratic Slope:	$\beta _1^{male} = \beta _1^{female}$ $\beta _2^{male} = \beta _2^{female}$ $\beta _3^{male} = \beta _3^{female}$	3.738	3	.291

*Note*: I = Intercept; S = Linear Slope; Q = Quadratic Slope; $\beta _1$ = norm acceptance $\rightarrow$ I; $\beta _2$ = norm acceptance $\rightarrow$ S; $\beta _3$ = norm acceptance $\rightarrow$ Q

**Table 15:** Results Bayesian Wald Test (School Type/Conditional Growth Model)

Hypothesis		$\chi ^2$	*df*	*p*
Equality of all regression coefficients:	$\beta _1^{GYM} = \beta _1^{RS} = \beta _1^{HS} = \beta _1^{GES}$ $\beta _2^{GYM} = \beta _2^{RS} = \beta _2^{HS} = \beta _2^{GES}$ $\beta _3^{GYM} = \beta _3^{RS} = \beta _3^{HS} = \beta _3^{GES}$	23.373	9	.005
Additional release of the regression coefficient from norm acceptance on I (GYM group):	$\beta _1^{RS} = \beta _1^{HS} = \beta _1^{GES}$ $\beta _2^{GYM} = \beta _2^{RS} = \beta _2^{HS} = \beta _2^{GES}$ $\beta _3^{GYM} = \beta _3^{RS} = \beta _3^{HS} = \beta _3^{GES}$	14.652	8	.066

*Note*: I = Intercept; S = Linear Slope; Q = Quadratic Slope; $\beta _1$ = norm acceptance $\rightarrow$ I; $\beta _2$ = norm acceptance $\rightarrow$ S; $\beta _3$ = norm acceptance $\rightarrow$ Q

## Footnotes

1 e.g. R-package *blavaan* (Merkle et al., 2021) or M*plus* (Muthén & Muthén, 1998-2017).

2 Such models can generally also be implemented in programs such as *jags* (Plummer, 2003) or *stan* (Carpenter et al., 2017). However, this requires in-depth knowledge of the implementation of structural equation models in the respective programs and can be challenging, especially for users who are unfamiliar with Bayesian statistics or latent growth curve modeling.

3 Vector $\tau _y$ is fixed to zero for identification purposes.

4 This also applies to the case of a Poisson distributed variable because this is a special case of the negative binomial distribution for $\delta \rightarrow 0$.

5 Alternative metrics of time can be specified in both equations and allow further possibilities to specify developmental processes (Biesanz et al., 2004; Bollen & Curran, 2006, p. 102).

6 The inverse gamma distribution is used when there is a single latent variable. When the number of latent variables exceeds two, the inverse Wishart distribution is used.

7 Strictly speaking, the Bayesian Wald test is not a Bayesian inference test but a frequentist test based on the results of an MCMC procedure. Nevertheless, the term proposed by the developers will be used in the following applications. The test is preferred below because its robustness has already been shown in some cases of non-normality of the data and provides a simple form of testing parameter restrictions (see for example Woller & Enders, 2024).

8 Group specific intercept vector $\tau _y^g$ is fixed to zero for identification purposes.

9 Because of the federalistic government system in Germany, the structure of the educational system varies across the states. The *Gymnasium* is comparable to a high school, the *Realschule* and *Hauptschule* are comparable to a grammar school. In some states the *Gesamtschule* were established to offer an easier possibility to change between school types.

10 The data set, M*plus* and R scripts are available online under https://osf.io/9jvn7/.

11 Given that this study is concerned with the development of delinquency since the commencement of the panel study, the initial wave was selected as the central point for coding. Consequently, the determination of the factor loading matrix $\Lambda$ is constrained by the conditions set forth in Eq. 12. However, in case of a different focus should the focus of the study differ, a distinct approach to determining the factor loading matrices, particularly the zeros, could be employed (see also Biesanz et al., 2004)

12 The description of the estimated posterior distributions of all parameters can be found in Table  A2 in the appendix.

13 The posterior distributions for count variables reported in each example are on a logarithmic scale. For an interpretation on a count scale, the respective posterior values have to be exponentiated. In contrast to frequentist estimation, this is not a problem when considering the corresponding *HDI*s, as these do not inherently assume a specific (symmetric) distribution. Due to manuscript limits, only the logarithmised posterior distributions are presented. However, interested readers can easily obtain the exponential posterior distributions using the provided R scripts. The relationship between the exponentiated posterior distributions is no longer linearly additive but multiplicative. Therefore, the interpretation of the exponentiated posterior distributions is different. An exponentiated value below 1 is a negative effect (or a decrease), of 1 is no effect, and above 1 is a positive effect (or an increase).

14 Recall that the fit of a Bayesian model is given by the *Posterior Predictive P-Value* (PPP). For identification of the latent variable, the factor loading of item t0722 is fixed at 1.

## Code availability

All M*plus* scripts used to estimate the models presented, as well as the R scripts used to generate the graphs and summary statistics presented, are available at the Open Science Framework: https://osf.io/9jvn7/.

## Declarations

### Open Practices Statement

We did not preregister the design and analysis of this study. Data and code for all examples are available at https://osf.io/9jvn7/.
